# Essential Role for CD30-Transglutaminase 2 Axis in Memory Th1 and Th17 Cell Generation

**DOI:** 10.3389/fimmu.2020.01536

**Published:** 2020-07-21

**Authors:** Akane S. Suzuki, Ryoji Yagi, Motoko Y. Kimura, Chiaki Iwamura, Kenta Shinoda, Atsushi Onodera, Kiyoshi Hirahara, Damon J. Tumes, Ryo Koyama-Nasu, Siiri E. Iismaa, Robert M. Graham, Shinichiro Motohashi, Toshinori Nakayama

**Affiliations:** ^1^Department of Medical Immunology, Graduate School of Medicine, Chiba University, Chiba, Japan; ^2^Department of Immunology, Graduate School of Medicine, Chiba University, Chiba, Japan; ^3^Institute for Global Prominent Research, Chiba University, Chiba, Japan; ^4^Centre for Cancer Biology, SA Pathology and University of South Australia, Adelaide, SA, Australia; ^5^Molecular Cardiology and Biophysics Division, Victor Chang Cardiac Research Institute, Sydney, NSW, Australia

**Keywords:** TG2, CD30, memory Th cell generation, Th17, Th1, memory precursor, airway inflammation

## Abstract

Memory helper T (Th) cells are crucial for secondary immune responses against infectious microorganisms but also drive the pathogenesis of chronic inflammatory diseases. Therefore, it is of fundamental importance to understand how memory T cells are generated. However, the molecular mechanisms governing memory Th cell generation remain incompletely understood. Here, we identified CD30 as a molecule heterogeneously expressed on effector Th1 and Th17 cells, and CD30^hi^ effector Th1 and Th17 cells preferentially generated memory Th1 and Th17 cells. We found that CD30 mediated signal induced Transglutaminase-2 (TG2) expression, and that the TG2 expression in effector Th cells is essential for memory Th cell generation. In fact, *Cd30*-deficiency resulted in the impaired generation of memory Th1 and Th17 cells, which can be rescued by overexpression of TG2. Furthermore, *transglutaminase-2* (*Tgm2*)-deficient CD4 T cells failed to become memory Th cells. As a result, T cells from *Tgm2*-deficient mice displayed impaired antigen-specific antibody production and attenuated Th17-mediated allergic responses. Our data indicate that CD30-induced TG2 expression in effector Th cells is essential for the generation of memory Th1 and Th17 cells, and that CD30 can be a marker for precursors of memory Th1 and Th17 cells.

## Introduction

Upon encountering foreign antigens, naïve CD4 T cells proliferate and differentiate into effector helper T (Th) cells (1–4). After the clearance of pathogens, the majority of effector Th cells die during the contraction phase, but a few Th cells survive and are maintained as memory Th cells (5–9). Memory Th cells undergo rapid cell division upon re-encountering the same antigens, and induce stronger responses by producing large amounts of cytokines and a variety of chemokines, recruiting various effector cells, such as neutrophils, macrophages, and CD8 T cells, into inflammatory sites (10–12). Furthermore, memory Th cells contribute to the maintenance of memory CD8 T cells (13, 14) and help B cells produce high-affinity immunoglobulins (15, 16). Thus, memory Th cells play crucial roles in protective immune responses, which efficiently eliminate pathogens that have previously been encountered. Although the importance of memory Th cells for long lasting protective immunity is well-established, memory Th cells are also involved in the pathogenesis of inflammation; such memory Th cells are designated as pathogenic memory Th cells. Long survival of pathogenic memory Th cells underlies the pathogenesis of chronic inflammatory disorders, including asthma, eosinophilic esophagitis, allergic rhinitis, and atopic dermatitis (1, 17–20).

Several signaling pathways that control memory Th cell-fate decisions have been reported (21–27). For instance, the TCR signal strength affects memory Th cell generation and survival (28); IL-2 signaling enhances the long-term survival of memory Th cells (29); and CD28-dependent costimulatory signaling during the priming phase is required for optimal memory Th cell generation (30). Upon antigen recognition, T cells undergo asymmetric cell division, which results in the generation of two daughter cells with different propensities for the development of memory or effector T cells, supporting the hypothesis that cell-fate decision occurs during priming (31, 32). We also have identified a memory Th cell precursor enriched subpopulation (CCR7^hi^CD137^lo^) in the context of lipid metabolism (33). However, the molecular mechanisms governing memory Th cell generation are still not fully understood, given that the identification of memory T cell precursors that are induced during immune responses remains problematic. In contrast, memory CD8 T cell precursors have been identified based on the expression of various cell-surface molecules, such as CD25, IL-7Rα, or KLRG1 (34–38).

We show here that CD30-induced TG2 expression in effector Th1 and Th17 cells is essential for memory Th cell generation. Specifically, we found that CD30^hi^ but not CD30^lo^ effector Th1 and Th17 cells readily become memory Th cells, whereas loss of CD30 prevents the differentiation of Th1 and Th17 cells into memory Th cells. Moreover, we found that CD30^hi^ Th cells express high levels of transglutaminase 2 (*Tgm2*), the expression of which is induced by CD30 signaling, and TG2 overexpression in *Cd30*-deficient Th cells restores memory Th cell generation. Taken together, these findings indicate that CD30-TG2 axis is an important signaling pathway for memory Th cell generation.

## Materials and Methods

### Mice

BALB/c and C57BL/6 mice were obtained from CLEA Japan Inc. *Cd30*^−/−^ and *Tgm2*^fl/fl^ mice were purchased from Jackson Laboratory, and Ly5.1 mice were purchased from Sankyo Laboratory. OVA-specific TCR-αβ (DO11.10) transgenic (Tg) mice were provided by Dr. Loh (Washington University School of Medicine, St. Louis, MO, USA) (39). For antigen-specific activation of CD4 T cells, mice expressing the OTII TCRαβ specific for residues 323–339 of OVA were also used (40). *Tgm2*^−/−^ mice were kindly provided by Dr. Soichi Kojima (Molecular Ligand Biology Research Team, Chemical Genomics Research Group, Chemical Biology Department, RIKEN Advanced Science Institute) (41, 42). All mice were maintained under specific pathogen-free conditions in the Chiba University animal facility and used at 6–12 weeks of age under an approved protocol according to the guidelines of Chiba University.

### *In vitro* Th Cell Differentiation and Adoptive Cell Transfer

Splenic CD4 T cells were isolated using an autoMACS Sorter (Miltenyi Biotec), and then naïve CD4 T cells (CD44^low^CD62L^high^) were further purified using a FACSAria cell sorter (BD Biosciences), yielding a purity of >98%. DO11.10 Tg or OTII Tg naïve CD4 T cells were stimulated with 0.3 μM OVA peptide (Loh15) together with irradiated (30.67 Gy) T cell-depleted splenocytes from BALB/c or C57BL/6 mice, respectively, for 6 days under the following conditions: for Th1 cell differentiation, 30 U/ml IL-2 culture sup, 100 U/ml IL-12 (Wako), and 1 μg/ml anti-IL-4 Ab (BioLegend): for Th2 cell differentiation, 30 U/ml IL-2 culture sup, 100 U/ml IL-4 (PeproTech), and 10 μg/ml anti-IFNγ Ab (BioLegend): for Th17 cell differentiation, 10 ng/ml IL-6 (PeproTech), and 10 ng/ml IL-1β (PeproTech), 10 ng/ml IL-23 (R&D), 10 μg/ml anti-IL-4 Ab, and 10 μg/ml anti-IFNγ Ab (BioLegend). *In vitro*-differentiated effector Th cells (5 × 10^6^ to 3 × 10^7^ cells) were intravenously transferred into BALB/c, C57BL/6, or BALB/c nu/nu recipient mice as indicated.

This adoptive transfer model to track CD4 memory T cells is a well-established system (43–45). Th cells recovered from the mice that were adoptively transferred with *in vitro* differentiated effector Th cells more than 1 month ago are reported to acquire memory signatures; i.e., expression of memory cell surface markers (CD44^hi^ CD62L^hi^ IL-7Rα^hi^), and the ability to proliferate rapidly and to produce large amounts of effector cytokines upon antigen stimulation (44).

### *In vitro* Proliferation Assay

Splenic naïve CD4 T cells were labeled with CFSE and then stimulated with OVA peptide together with irradiated T cell-depleted splenocytes under Th1 or Th17 conditions for 3 or 4 days. In some experiments, DO11.10 Tg splenic naïve CD4 T cells were stimulated with immobilized anti-TCRβ Ab (3 μg/ml) plus anti-CD28 Ab (1 μg/ml) under Th1 conditions as indicated.

### Immunofluorescent Staining for Flow-Cytometric Analyses

The antibodies used for the detection of surface and intracellular molecules are listed in the supplementary experimental procedures (Supplemental Table 1). Flow cytometry data were acquired on a FACSCantoII (BD Biosciences) using the FACSDiva software program (BD Biosciences) and analyzed using the FlowJo software program (Tree Star). Intracellular staining was performed as previously described (46). In brief, the *in vitro* differentiated Th cells were re-stimulated with 10 ng/ml phorbol 12-myristate 13-acetate (PMA) and 500 nM ionomycin in the presence of 2 μM monensin for 4 h. These stimulated cells were first stained the cell surface molecules such as CD4 and CD44, fixed with 4% paraformaldehyde for 10 min at room temperature, permeabilized in PBS containing 0.5% Triton X-100 and 0.1% BSA, then stained for cytokines, and were analyzed by FACSCantoII. The data were analyzed using FlowJo software program (BD Biosciences) and analyzed using the FlowJo software program (Tree Star).

### RNA Sequencing Analyses

Total RNA was extracted with TRIzol reagent (Life Technologies) according to the manufacturer's instructions. For cDNA library construction, we used TruSeq RNA Sample Prep Kit v2 (Illumina) according to the manufacturer's protocol. Sequencing the library fragments was performed on the HiSeq 1500 System. For the data analysis, read sequences (50 bp) were aligned to the mm 10 mouse reference genome (University of California, Santa Cruz [UCSC], December 2011) using the Bowtie (version 0.12.8) and TopHat (version 1.3.2) software programs. Fragments per kilobase of exon per million mapped reads (FPKM) for each gene were calculated using Cufflinks (version 2.0.2).

### Quantitative Reverse Transcription-Polymerase Chain Reaction (qRT-PCR)

Total RNA was isolated using TRIzol reagent. cDNA was synthesized using oligo (dT) primer and Superscript II RT (Life Technologies). qRT-PCR was performed using an ABI PRISM 7500 Sequence Detection System. Data are shown relative to β-actin. The probes for the detection of genes in this study were purchased from Roche Applied Science. The probes and primers are listed in Supplemental Table 2.

### Microarray Data Collection and Analyses

Total RNA was isolated using TRIzol reagent (Life Technologies). RNA was labeled with a 3′ IVT Express kit (Affymetrix) and hybridized to GeneChip Mouse Genome 430 2.0 arrays (Affymetrix) according to the manufacturer's protocols. The expression values were determined with the GeneChip Operating Software (GCOS) program (Affymetrix).

### CD30 Stimulation With Recombinant CD153

*In vitro* stimulation using recombinant CD153 was performed as previously reported (47). In brief, BALB/c or C57BL/6 naïve splenic CD4 T cells were stimulated with immobilized anti-TCRβ Ab (3 μg/ml) plus anti-CD28 Ab (1 μg/ml) under Th1 or Th17 conditions (described above in the section of *in vitro Th cell differentiation and adoptive cell transfer*) for 24 h and then further stimulated with immobilized and soluble recombinant mouse CD153 (500 ng/ml, R&D) for further 4 days.

### Knockdown and Overexpression Assays

For knockdown assay, non-targeting control or *Tgm2* shRNA from a pMLP retroviral vector (Trans Omic) were inserted into a pLMP retroviral vector (Open Biosystems). Naïve CD4 T cells were cultured for 2 days under Th1 conditions and then infected with retrovirus vector containing non-targeting control shRNA (pLMP-GFP) or *Tgm2* shRNA (pLMP-sh*Tgm2*-GFP). Three days after the infection, the GFP-positive infected cells were purified by cell sorting, and used for further analyses. For overexpression assay, naïve CD4 T cells were cultured for 1 day under Th1 conditions and then infected with retrovirus vector pMXs-Mock- hNGFR or pMXs-*Tgm2*-hNGFR. Four days after the infection, hNGFR^+^ infected cells were purified by cell sorting, and used for further analyses.

### *In vivo* Immunization

The mice transferred with splenic CD4 T cells (1 × 10^6^ cells) from OTII Tg *Tgm2*^+/+^ or *Tgm2*^−/−^ mice were intraperitoneally injected with 100 μg NP_19_-OVA (Biosearch Technologies) in CFA (Sigma) next day after cell transfer. Serum samples were collected 1 and 3 weeks after the immunization. The concentrations of NP-specific Igs (IgG1 and IgG2c) were determined by ELISA (48). Alternatively, *Tgm2*^fl/fl^ *Cd4Cre* and control *Tgm2*^fl/fl^ mice were intraperitoneally injected with 100 μg whole OVA (Sigma) plus 10 μg LPS (Invivogen) in 200 μl PBS at an interval of 2 weeks. Serum samples were collected and analyzed 4 weeks after the last immunization. To detect *in vivo* generated memory Th cells, whole splenocytes were stimulated with OVA (100 μg /mL) for 4 h in the presence of 2 μM monensin, and detected CD154^+^ and IFNγ^+^ CD4T cells by intracellular staining (49).

### Induction of Th17 Cell Dependent Airway Inflammation *in vivo*

*In vitro*-differentiated OTII Tg *Tgm2* WT and KO effector Th17 cells (1 × 10^7^ cells) were transferred intravenously into C57BL/6 mice. Three weeks later, the mice were challenged with intranasal administration of OVA (dissolved in PBS, 100 μg per 30 μl) on days 0, 1 and 2, and analyzed on day 3 to induce Th17 cell dependent airway inflammation.

### Measurement of AHR

The respiratory parameters were obtained from the mice under the exposures of incremental doses of aerosolized methacholine (0, 6, 12, 24, 48, and 96 mg/ml in saline). The degree of AHR was assessed by a computer-controlled small animal ventilator (SCIREQ) (50).

### Collection of BAL fluid

A total of 100,000 viable BAL cells was cytocentrifuged onto slides by a Cytospin 4 (Thermo Electron) and stained with May-Grunwald-Giemsa solution (Merck). Two hundred leukocytes were counted on each slide. The cell types were identified using morphological criteria. The concentration of IL-17A, IL-1β, and IL-6 in the BAL fluid was assessed with a CBA Mouse Enhanced Sensitivity Flex Set System (BD Biosciences).

### Lung Histology and Immunohistochemistry

The lungs were fixed in 10% (v/v) formalin, sectioned and stained with hematoxylin and eosin (H&E) for the examination of pathological changes under a light microscope at 200×.

### Statistical Analyses

Unless otherwise indicated, *p*-values were calculated using Student's *t*-test or a two-way analysis of variance.

## Results

### Memory Th1 and Th17 Cells Are Preferentially Generated From CD30^hi^ Effector Th Cell

To identify cell surface markers expressed on memory-precursor effector CD4 T cells, we first performed RNA sequencing analysis with *in vitro*-differentiated Th1, Th2, Th17 cells and naïve CD4 T cells (Supplemental Figure 1A). We found 10 genes classified as receptors that were highly expressed on all effector Th subsets compared to naïve CD4 T cells (Supplemental Figure 1B). Flow cytometry analysis showed that effector Th1 and Th17 cells exhibited striking heterogeneity in the expression of TNFRSF8 (CD30); whereas, LILRB4 (CD85k), leukotriene B4 receptor 1 (LTB4R1), TNFRSF9 (CD137), IL-2Rα (CD25), IL-2Rβ (CD122), Integrin alpha 7 (ITGA7), TNFRSF4 (OX40), and TNFRSF18 (GITR) were uniformly expressed on all Th cells (Supplemental Figures 1C,D). We found that the CD30-expressing CD4 T cells gradually appeared between 2 and 4 days after stimulation (Supplemental Figure 1E), and that CD30 molecules were heterogeneously expressed on Th1 and Th17 cells. We considered the possibility that CD30 expression could be used for the distinction between short-lived effector Th cells and long-lived memory Th cell precursors.

**Figure 1:**
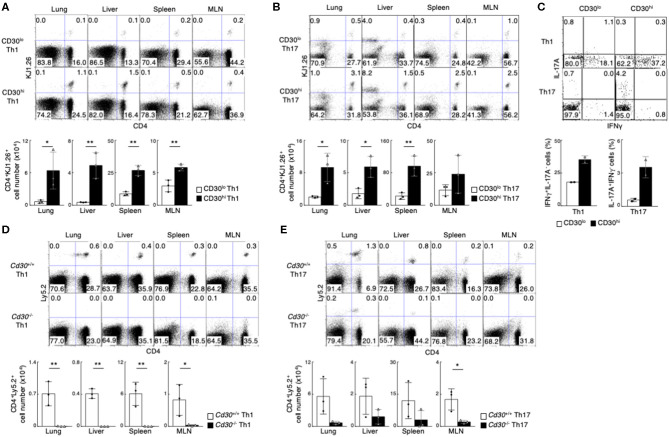
Preferential generation of memory Th1 and Th17 cells from effector CD30^hi^ Th cells. **(A**,**B)** *in vitro*-differentiated DO11.10 Tg CD30^hi^, or CD30^lo^ Th1 **(A)** or Th17 **(B)** cells (5 × 10^6^) were transferred into BALB/c mice, and the mice were analyzed 1 month later (the protocol shown in Supplemental Figure 1F). Representative CD4/KJ1.26 profiles of the transferred cells in the lung, liver, spleen, and mesenteric lymph node (MLN) (upper panels) and the absolute cell numbers of CD4^+^KJ1.26^+^ transferred cells in three individual animals are shown (lower panels). Data are representative of three independent experiments. **(C)** Cytokine profiles of splenic CD4^+^KJ1.26^+^ memory Th1 and Th17 cells recovered from the host mice as in **(A,B)** are shown. Whole splenocytes were restimulated with PMA + Ionomycin for 4 h in the presence of monensin, and performed intracellular staining to detect the indicated cytokines. **(D,E)** *in vitro*-differentiated Th1 **(D)** or Th17 **(E)** cells (3 × 10^7^) from OTII Tg *Cd30*^+/+^ *or Cd30*^−/−^ mice (Ly5.2) were transferred into Ly5.1 mice and analyzed 1 month later (the protocol shown in Supplemental Figure 1F). Representative CD4/Ly5.2 profiles of the transferred cells in the indicated tissues (upper panels) and the absolute cell numbers of CD4^+^Ly5.2^+^ transferred cells are shown (lower panels). Data are representative of at least two independent experiments. Mean values with SDs (*n* = 3) are shown (**P < 0.01, *P < 0.05).

To test this possibility, we decided to examine if there were any different abilities of CD30^hi^ and CD30^lo^ effector Th cells to generate memory Th cells by using an adoptive transfer system (51). CD30^hi^ and CD30^lo^ effector Th1 cells with ovalbumin (OVA) peptide antigen-specific TCR (DO11.10 Tg mice) were prepared *in vitro*, isolated by cell sorting and each cell type was separately transferred into the syngeneic BALB/c mice (Supplemental Figure 1F). One month later, we examined the frequency of transferred Th1 cells using anti-DO11.10 TCR antibody (clone: KJ1.26). We found that the number of CD4^+^KJ1.26^+^ memory Th1 cells in various organs (lung, liver, spleen and mesenteric lymph nodes [MLNs]) was much higher in the mice transferred with effector CD30^hi^ Th1 cells than the mice with effector CD30^lo^ Th1 cells (Figure 1A). The same results were obtained in the case of using CD30^hi^ and CD30^lo^ Th17 cells (Figure 1B). We next examined the function of CD4^+^KJ1.26^+^ memory Th1 and Th17 cells by stimulating them *in vitro* to detect their cytokine productions (Figure 1C). The frequencies of IFNγ-producing memory Th1 cells and IL-17A-producing memory Th17 cells were significantly reduced in the cells derived from CD30^lo^ effector Th cells as compared to CD30hi effector Th cells. These results indicate that CD30^hi^ effector Th cells are more susceptible to become memory Th cells than CD30^lo^ effector Th cells, and that the precursors of memory Th1 and Th17 cells appear to be enriched in the population of CD30^hi^ effector Th cells.

### CD30-Deficient Effector Th Cells Failed in the Generation of Memory Th Cell

We further examined whether CD30 expression itself is important for memory Th cell generation. For this purpose, we used CD4 T cells from OTII TCR Tg *Cd30*^−/−^ (Ly5.2) and *Cd30*^+/+^ (C57BL/6, Ly5.2) mice. *In vitro* differentiated *Cd30*-deficient and *Cd30*-sufficient Th1 or Th17 cells expressing OTII TCR were adoptively transferred into Ly5.1 host mice (Supplemental Figure 1G), and these mice were analyzed 1 month later. We found that memory Th1 and Th17 cell generation in the mice transferred with *Cd30*-deficient effector Th cells was diminished (Figures 1D,E). These data demonstrate that CD30 expression on Th cells is required for the generation of memory Th1 and Th17 cells.

### TG2 Is Induced by CD30-Mediated Signaling in Th Cells

**Figure 2:**
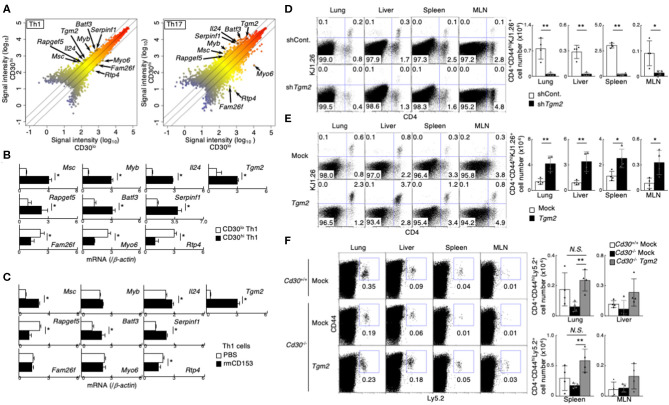
Importance of CD30 signaling-induced Transglutaminase 2 (TG2) expression on memory Th1 and Th17 cell generation. **(A)** Scatterplot showing the correlation of gene expression in cDNA microarray analysis between CD30^hi^ vs. CD30^lo^ cells in Th1 (left) and Th17 (right) cells. Signal intensities (log_10_) are shown. Differentially expressed genes were selected using the following criteria: (1) signal “present” call and (2) signal intensity >= 300 for higher expressed genes. (3) 2-1.5-fold change in gene expression. **(B)** mRNA expression of the indicated genes between CD30^hi^ and CD30^lo^ Th1 cells by qRT-PCR. Mean values with SDs are shown (*P < 0.01). **(C)** mRNA expression of the indicated genes after recombinant mouse CD153 stimulation. Naïve CD4 T cells were cultured under Th1 cell conditions for 24 h and the cells were treated with or without recombinant mouse CD153 (the protocol shown in Supplemental Figure 2C). Mean values with SDs are shown (*P < 0.01). **(D,E)** Effects of TG2 knockdown **(D)** and overexpression **(E)** on the generation of memory Th cells. DO11.10 Tg Th1 cells (the protocol shown in Supplemental Figure 2E). TG2 knockdown **(D)** and overexpressed **(E)** cells transferred into the host mice were detected based on their CD4 and KJ1.26 expression (left panels), and the absolute cell numbers of CD4^+^CD44^hi^KJ1.26^+^ transferred cells are shown (right panels). Mean values with SDs (*n* = 4) are shown (**P < 0.01, *P < 0.05). Data are representative of at least two independent experiments. **(F)** *Tgm2* or Mock retrovirus-transduced Ly5.2 *Cd30* sufficient or deficient Th1 cells (5 × 10^6^) were transferred into Ly5.1 mice, and these mice were analyzed 1 week later as shown in Supplemental Figure 2E. A representative Ly5.2/CD44 profile of the transferred CD4 T cells in the indicated tissues (left panels) and the absolute cell numbers of CD4^+^CD44^hi^Ly5.2^+^ transferred cells (right panels) are shown. Mean values with SDs (*n* = 4) are shown (**P < 0.01, *P < 0.05). Data are representative of at least two independent experiments.

To identify the molecules that were differentially expressed in CD30^hi^ and CD30^lo^ effector Th1 and Th17 cells, we next performed a microarray analysis and compared gene expression between CD30^hi^ and CD30^lo^ effector Th cells. We found that 175 genes were differentially expressed between CD30^hi^ and CD30^lo^ Th1 cells, and 607 genes were differentially expressed between CD30^hi^ and CD30^lo^ Th17 cells. Among them, 10 genes were commonly shared in both cases; 7 out of 10 genes had higher expression and 3 genes had lower expression in CD30^hi^ cells than in CD30^lo^ cells (Figure 2A, Supplemental Figure 2A). Further, we confirmed mRNA expression of these genes between CD30^hi^ and CD30^lo^ effector Th1 and Th17 cells by quantitative RT-PCR (Figure 2B, Supplemental Figure 2B). Since CD30 is known as a receptor that can signal, we next examined whether the expression of these 10 genes was directly regulated by CD30-mediated signaling. To do so, *in vitro* differentiated Th1 or Th17 cells were stimulated with soluble recombinant mouse CD153 (rmCD153), the CD30 ligand, for 4 days, and mRNA expression of these genes was analyzed (Supplemental Figure 2C). We found that rmCD153 stimulation increased the mRNA expression of *Tgm2, Musculin* (*Msc*), *serine peptidase inhibitor, clade F, member 1* (*Serpinf1*), and *Basic leucine zipper transcription factor, ATF-like3* (*Batf3*), in both Th1 and Th17 cells (Figure 2C, Supplemental Figure 2D). Among them, *Tgm2* expression was remarkably increased approximately 8- and 6-fold change in Th1 and Th17 cells, respectively; we therefore decided to analyze in more detail the function of TG2 (encoded by *Tgm2*) in the generation of memory Th1 and Th17 cells.

### TG2 Is Important for Memory Th Cell Generation

To determine whether TG2, encoded by *Tgm2* gene, is involved in memory Th generation, we made TG2-knockdown Th1 cells by retrovirally introducing short hairpin RNA (shRNA) specific for *Tgm2*. TG2-knockdown Th1 cells were isolated by cell sorting based on their GFP reporter expression, and then were transferred into BALB/c *nu/nu* mice (Supplemental Figures 2E,F). One week after the transfer, we assessed the number of CD4^+^CD44^hi^KJ1.26^+^ effector Th1 cells in various tissues. Notably, the number of TG2-knockdown Th1 cells was significantly decreased compared to that of control shRNA-transduced Th1 cells in all tissues that we examined (Figure 2D).

We next examined the impact of overexpression of TG2 on memory Th cell generation. TG2 was retrovirally introduced into *in vitro*-differentiated effector Th1 cells, the TG2-introduced Th cells were isolated based on the expression of human nerve growth factor receptor (hNGFR), and then transferred into BALB/c *nu/nu* mice (Supplemental Figures 2E,G). We found that the TG2-overexpresing Th1 cells increased cell recovery in the host mice (Figure 2E), indicating that TG2 is important in memory Th1 cell generation.

### TG2 Overexpression Rescues Memory Th Cell Generation of Cd30-Deficient Th Cells

We next examined whether the impairment of memory Th cell generation in *Cd30*-deficiency can be restored by the over expression of TG2. To do so, *Tgm2* or mock retrovirus-transduced *Cd30*-sufficient or *Cd30*-deficient Th1 cells were transferred into Ly5.1 mice, and the mice were analyzed a week later. Notably, we found that overexpression of TG2 in *Cd30*-deficient Th1 cells significantly improved their recovery in the lung and spleen (Figure 2F), demonstrating that TG2 plays an important role for memory Th1 cell generation. These results indicate that TG2 is a downstream target molecule of CD30 signaling, and that the CD30-TG2 axis is critical for the generation of memory Th cells.

### TG2 Is Required for the Generation of Memory Th1 and Th17 Cells

**Figure 3:**
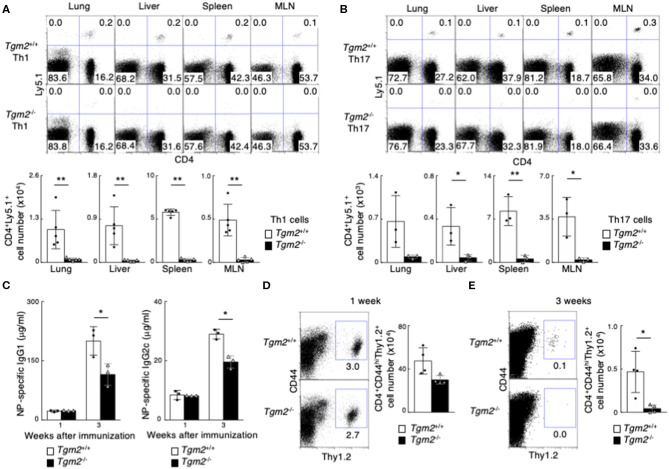
Failure of memory Th1 and Th17 cell generation in the absence of TG2. **(A,B)** *in vitro*-differentiated Th1 **(A)** or Th17 **(B)** cells (3 × 10^7^) from Ly5.1 OTII Tg *Tgm2*^+/+^ or *Tgm2*^−/−^ mice were transferred into C57BL/6 (Ly5.2) host mice, and these mice were analyzed 1 month later, as described in Supplemental Figure 3E. Representative CD4/Ly5.1 profiles of the transferred cells in the indicated tissues (upper panels) and the absolute cell numbers of CD4^+^Ly5.1^+^ transferred cells are shown (lower panels). Mean values with SDs (*n* = 3–5) are shown (**P < 0.01, *P < 0.05). Data are representative of at least two independent experiments. **(C–E)** Thy1.2 splenic CD4 T cells (1 × 10^6^) isolated from OTII Tg *Tgm2*^+/+^ or *Tgm2*^−/−^ mice were transferred into Thy1.1 C57BL/6 mice, and the mice were immunized with 100 μg NP-OVA in CFA, as described in Supplemental Figure 3H. **(C)** Concentrations of anti-NP-IgG1 and anti-NP-IgG2c antibodies in sera at each time point after the immunization was analyzed by ELISA assays. Mean values with SDs (*n* = 3) are shown (*P < 0.05). **(D,E)** Representative Thy1.2/CD44 profiles of the transferred cells in the spleen (left panels) and the absolute cell numbers of CD4^+^CD44^hi^Thy1.2^+^ transferred cells (right panels) 1 week **(D)** or 3 weeks **(E)** after cell transfer are shown. Mean values with SDs (*n* = 4) are shown (*P < 0.05).

To clarify the role of TG2 in memory Th cell generation *in vivo*, we next used *Tgm2*^−/−^ mice in which the TGase catalytic core domain was deleted (41). *Tgm2*^−/−^ mice had normal CD4/CD8 thymocyte and splenocyte profiles (Supplemental Figure 3A), with comparable cell-surface expression of CD3ε, TCRβ, CD25, CD122, CD44, CD62L, and CD69 on splenic CD4 T cells (Supplemental Figure 3B). *In vitro* antigen-induced cell division of *Tgm2-*deficient Th cells under Th1 and Th17 cell conditions was similar to that of WT Th cells (Supplemental Figure 3C), and *in vitro* differentiation to Th1 and Th17 cells was comparable between *Tgm2-*deficient and -sufficient cells (Supplemental Figure 3D). Given these findings, *Tgm2-*deficient CD4 T cells appear to develop normally in the thymus and have no obvious defects in effector Th cell differentiation *in vitro*. We then prepared *in vitro*-differentiated Ly5.1 OTII Tg *Tgm2*-sufficient and -deficient effector Th1 and Th17 cells, separately transferred them into syngeneic C57BL/6 (Ly5.2) mice, and then analyzed the mice 1 month later (Supplemental Figure 3E). Of note, the number of CD4^+^Ly5.1^+^ memory Th cells was substantially lower in the mice given *Tgm2*-deficient effector Th1 or Th17 cells than in the mice given *Tgm2*-sufficient effector Th cells (Figures 3A,B). These results indicate that TG2 expression is required for the optimal generation of memory Th1 and Th17 cells.

In contrast to the expression of CD30 on effector Th1 and Th17 cells, effector Th2 cells uniformly expressed CD30 (Supplemental Figure 1C). We also found that effector Th2 cells expressed more *Tgm2* mRNA than CD30^low^ Th1 and Th17 cells, and the expression level of *Tgm2* in effector Th2 cells was almost equivalent to that in CD30^hi^ effector Th1 or Th17 cells (Supplemental Figures 2B,H, Figure 2B). Interestingly, *Tgm2*-deficiency in Th2 cells did not influence the number of memory Th2 cells in our experimental systems (Supplemental Figures 3F,G), indicating that TG2 is not involved in the generation of memory Th2 cells.

### TG2 Is Required for the Acquired Immune Response *in vivo*

To further evaluate the role of TG2 in memory Th cell generation, we next examined *in vivo* immune responses in the mice with *Tgm2*-deficient CD4 T cells compared to the mice with *Tgm2*-sufficient WT CD4 T cells. We transferred Thy1.2 OTII *Tgm2*-sufficient or -deficient CD4 T cells into congenic Thy1.1 host mice, and then the mice were immunized with NP_19_-coupled OVA plus CFA to generate memory Th cells *in vivo* (25) (Supplemental Figure 3H). The concentration of antigen-specific IgG1 and IgG2c antibodies in sera 1 week after immunization was comparable between both mice; however, the mice with *Tgm2*-deficient cells showed significantly lower amounts of antigen-specific antibody in sera 3 weeks after immunization (Figure 3C), with a decreased number of *Tgm2*-deficient effector CD4 T cells in the spleen, 3 weeks after immunization (Figures 3D,E). These results therefore indicate that TG2 is required for memory Th cell generation and plays important roles in the *in vivo* production of antigen-specific antibodies.

### Memory Th17-Driven Allergic Airway Inflammation Is Attenuated in Mice With Tgm2 Deficient Th17 Cells

**Figure 4:**
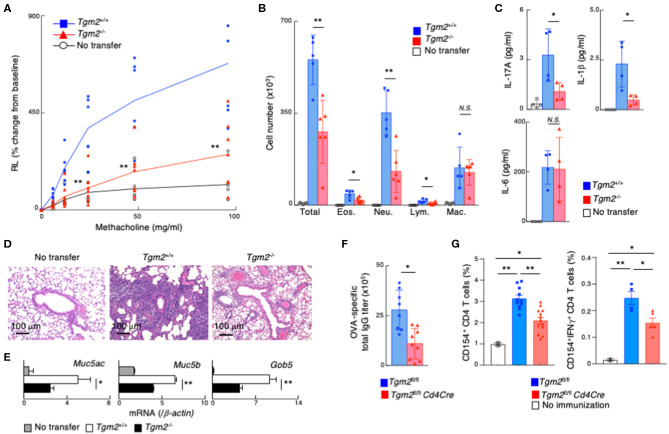
Impairment of memory Th17-driven allergic airway inflammation and antibody production in mice with TG2 deficient T cells. **(A–E)** *in vitro-*differentiated OTII Tg *Tgm2*^+/+^ or *Tgm2*^−/−^ Th17 cells (1 × 10^7^) were transferred into C57BL/6 mice, and 3 weeks later, the mice were challenged intranasally with OVA for three consecutive days (the protocol described in Supplemental Figure 4A). **(A)** AHR in response to increasing doses of methacholine was assessed 1 day after the last OVA challenges. Mean values with SDs (*n* = 6–7) are shown. **(B)** The number of total infiltrated mononuclear cells (Total), eosinophils (Eos.), neutrophils (Neu.), lymphocytes (Lym.), and macrophages (Mac.) in the BAL fluid. Mean values with SDs (*n* = 5) are shown. **(C)** The concentrations of IL-17A, IL-1β, and IL-6 in the BAL fluid measured by a cytometric bead array (CBA). Mean values with SDs (*n* = 4) are shown. **(D)** Histological analysis (H&E staining) of the lung. **(E)** *Muc5ac, Muc5b*, and *Gob5* mRNA expressions in the lung. **(F,G)** Mice were immunized with 100 μg OVA plus 10 μg LPS twice at an interval of 2 weeks, and the blood samples were taken 4 weeks after the last immunization **(F)**. The splenocytes were stimulated with whole OVA *in vitro* for 4 h and analyzed the frequencies of CD154^+^ cells and CD154^+^IFNγ^+^ cells **(G)** (the protocol described in Supplemental Figure 4C) (*n* = 5–7). Data are representative of at least two independent experiments (**P < 0.01, *P < 0.05).

We investigated TG2 function in memory Th17-dependent allergic airway inflammation, which is known to be associated with increased airway hyperreactivity, increased airway neutrophils, and mucous cell metaplasia in airway epithelial cells (52). *In vitro*-differentiated OTII Tg *Tgm2*-sufficient or -deficient effector Th17 cells were transferred into C57BL/6 mice, and 3 weeks later these mice were challenged with intranasal administration of OVA to induce airway inflammation (Supplemental Figure 4A). Methacholine-induced airway hyperresponsiveness (AHR) was significantly decreased in the mice with *Tgm2*-deficient Th17 cells compared to the mice with *Tgm2*-sufficient WT Th17 cells (Figure 4A). The total number of infiltrating leukocytes and neutrophils in the bronchoalveolar lavage (BAL) fluid was also decreased in the mice with *Tgm2*-deficient Th17 cells compared to the mice with *Tgm2*-sufficient WT Th17 cells (Figure 4B). The amounts of IL-17A and IL-1β in the BAL fluid were significantly decreased in the mice with *Tgm2*-deficient Th17 cells compared to the mice with *Tgm2*-sufficient WT Th17 cells, whereas the amount of IL-6 was comparable in the both mice (Figure 4C). Furthermore, the mice with *Tgm2*-deficient Th17 cells showed diminished infiltration of mononuclear cells in the peribronchiolar and perivascular regions of the lungs (Figure 4D), and less mRNA expression of *Muc5ac, Muc5b*, and *Gob5*, which are molecular markers for mucus production and goblet cell hyperplasia, than the mice with *Tgm2*-sufficient WT Th17 cells (Figure 4E). These results indicate that *Tgm2*-deficiency in effector Th17 cells results in impaired antigen-specific recall inflammatory responses.

### TG2 Is Crucial for Memory Th Cell Generation *in vivo*

Finally, we assessed the role of TG2 on *in vivo* memory Th cell generation in non-adoptive transfer experimental models. To do so, we generated *Tgm2*^fl/fl^ *Cd4Cre* mice that lack the *Tgm2* gene specifically in T cells. CD4/CD8 profiles of thymocytes and splenocytes from *Tgm2*^fl/fl^ *Cd4Cre* mice were normal, with comparable cell-surface expression of CD3ε, TCRβ, CD25, CD122, CD44, CD62L, and CD69 on splenic CD4 T cells (Supplemental Figure 4B). *Tgm2*^fl/fl^ *Cd4Cre* mice were immunized with OVA plus LPS twice and the mice were analyzed 4 weeks after the last immunization (Supplemental Figure 4C). The production of antigen-specific total IgG antibodies in sera was significantly lower in *Tgm2*^fl/fl^ *Cd4Cre* mice than control *Tgm2*^fl/fl^ mice (Figure 4F). To detect antigen-specific memory CD4T cells, we stimulated splenocytes with OVA antigen *in vitro* for 4 h and analyzed CD40 ligand (CD40L, CD154) expression (49). The frequency of CD40L expressing splenic CD4 T cells as well as IFNγ producing CD40L-expressing CD4 T cells after OVA stimulation was significantly lower in *Tgm2*^fl/fl^ *Cd4Cre* mice than control *Tgm2*^fl/fl^ mice (Figure 4G, Supplemental Figure 4D), demonstrating the decreased number of antigen-specific memory T cells in *Tgm2*^fl/fl^ *Cd4Cre* mice. These results indicate that TG2 is required for *in vivo* memory Th cell generation.

## Discussion

In the present study, we showed that CD30-TG2 axis plays a critical role in the generation of memory Th1 and Th17 cells. *Cd30*-deficient effector Th1 and Th17 cells failed to efficiently generate memory Th1 and Th17 cells, whereas overexpression of TG2 in *Cd30*-deficient effector Th cells restored memory Th1 and Th17 cell generation. We found that *Tgm2* mRNA was highly expressed in CD30^hi^ effector Th cells and that *Tgm2* expression was induced by CD30 signaling. Furthermore, the loss of TG2 resulted in a failure to generate memory Th cells *in vivo* and in a decrease in the levels of antigen-specific antibodies. In addition, *Tgm2*-deficiency resulted in impaired inflammatory responses in a Th17-mediated airway inflammation model, and impaired antigen-specific antibody production. Thus, the CD30-TG2 axis is essential for the optimal generation of memory Th1 and Th17 cells.

It was previously reported that CD30 signaling is required for memory T cell generation (53–55), in which CD30- and OX40-dependent signals synergistically play important roles in the generation of memory Th cells through cell-cell interaction. However, how CD30 signaling contributes to memory Th cell generation was not addressed. Our study identifies that TG2 is the downstream molecule that is induced by CD30 signaling during immune responses, and that TG2 expression is essential for memory Th cell generation.

TG2 is ubiquitously expressed in numerous tissues and is also broadly distributed in cell organelles, including cytoplasmic portion, nuclear membrane, mitochondria, and extracellular matrix components (56–59). TG2 is a multifunctional enzyme with protein cross-linking activities and also works as a GTPase (60, 61). Thus, TG2 is implicated in the control of a variety of cellular processes, including signal transduction, cell adhesion and spreading, wound healing, anti- and pro-apoptosis, and bone ossification (59, 62, 63). A recent study has reported that *Tgm2*-deficiency in CD8 T cells causes down-regulation of IL-7Rα, which is important for maintenance of memory CD8 T cells (64). Interestingly, it has been also reported that there is a correlation between TG2 expression and malignancy of cancer cells, and that TG2 is one of the target genes of hypoxia-inducible factor (23)-1α, which is a transcription factor induced under hypoxic conditions (65). In fact, overexpression of TG2 in a tumor cell line under hypoxic conditions significantly increases the number of cells compared to controls (66). During antigen recognition and T cell expansion, effector T cells also experience hypoxic conditions (67). Thus, effector Th cells, which acquire TG2 expression through the CD30-signaling pathway, may selectively survive and preferentially become memory Th cells.

We found that TG2 was involved in the generation of memory Th1 and Th17 cells but not memory Th2 cells. Both Th1 and Th17 cells are involved in the pathogenesis in chronic inflammation such as experimental autoimmune encephalomyelitis; therefore, it may be reasonable to have a common mechanism for generating memory Th1 and Th17 cells. In contrast, it is also known that Th1 and Th2 cells strongly antagonize the proliferation and differentiation of each other (1, 68). We assume that the immune system may utilize a different mechanism in the generation of memory Th2 cells from that of memory Th1/Th17 cells.

In summary, we have identified CD30 as a functional cell-surface molecule in effector Th cells to induce TG2 expression and facilitate the generation of memory Th1 and Th17 cells. CD30 can be used as a cell surface marker to identify a memory precursor population in effector Th1 and Th17 cells. The CD30-TG2 axis plays an important role in the generation of memory Th1 and Th17 cells, and secondary immune responses such as antigen-specific antibody production and Th17-mediated allergic airway inflammation. Thus, our findings provide new insights into the molecular mechanisms that govern memory Th cell generation and define TG2 as a novel therapeutic target for the treatment of Th17-mediated chronic airway inflammation.

## Data Availability Statement

The datasets presented in this study can be found in online repositories. The names of the repository/repositories and accession number(s) can be found below: https://www.ncbi.nlm.nih.gov/geo/, GSE151301; https://www.ncbi.nlm.nih.gov/geo/, GSE151691.

## Ethics Statement

The animal study was reviewed and approved by Chiba University.

## Author Contributions

AS and RY designed and did experiments and analyzed data. CI, KS, AO, KH, DT, RK-N, and SM did experiments and analyzed data. AS, RY, and MK interpreted the data and wrote the manuscript. SI and RG provided the mice and helpful discussion. TN directed the study, interpreted the data, and edited the manuscript. All authors contributed to the article and approved the submitted version.

## Conflict of Interest

The authors declare that the research was conducted in the absence of any commercial or financial relationships that could be construed as a potential conflict of interest. The reviewer LP declared a past co-authorship with one of the authors MK to the handling Editor.

## Supplementary Material

Supplemental Figure 1 CD30 expression in naïve CD4T, effector Th1, Th2 and Th17 cells. **(A)** A schematic illustration of the experimental protocol for *in vitro*-differentiated effector Th1, Th2, and Th17 cells. **(B)** Venn diagrams showing the overlap of “receptor” genes differentially expressed in Th1, Th2, and Th17 cells vs. naïve CD4 T cells. Gene expression in each Th cell population was measured by RNA-seq as FPKM (fragments per kilobase of exon per million mapped reads). Up-regulated genes were selected using the following criteria: (1) absolute FPKM > 50 in effector Th1, Th2, or Th17 cells, and (2) 2-fold change in the expression. “Receptor” (SP_PIR_Keyword) genes were selected using the DAVID analysis tool (http://david.abcc.ncifcrf.gov/home.jsp). The shared genes among Th1, Th2, and Th17 cells are listed (table). **(C,D)** Expression pattern of the indicated cell-surface marker molecules on naïve CD4 T cells, effector Th1, Th2, and Th17 cells 6 days after stimulation **(C)**. The frequency of CD30^hi^ and CD30^lo^ Th1, Th2, and Th17 cells were shown **(D)**. **(E)** The percentages of CD30-expressing cells during effector Th cell differentiation. DO11.10 Tg naïve CD4 T cells were stimulated as shown in **(A)**, and the percentages of CD30-expressing cells were measured at days 0, 1, 2, 4, and 6. **(F)** A schematic illustration of the experimental protocol for memory Th cell generation by adoptive transfer of DO11.10 Tg effector Th1 or Th17 cells, including the gating and sorting strategy of CD30hi and CD30lo Th cells. **(G)** A schematic illustration of the experimental protocol for memory Th cell generation using OTII Tg *Cd30*^+/+^ or *Cd30*^−/−^ naïve CD4 T cells (Ly5.2) Data are representative of at least two independent experiments **(B–D)**.

Supplemental Figure 2 Identification of the candidate genes that regulate memory Th1 and Th17 cell generation through CD30 signaling. **(A)** Venn diagrams of genes with changed expressions of a log_2_ value >1.5 between the CD30^hi^ and CD30^lo^ populations, measured by cDNA microarray analysis. Ten genes commonly changed expressions in Th1 and Th17 cells are listed (table). **(B)** mRNA expressions in CD30^hi^ and CD30^lo^ Th17 cells measured by qRT-PCR. **(C)** A schematic illustration of the experimental protocol for *in vitro* CD30 stimulation using recombinant mouse CD153 (CD30 ligand). **(D)** mRNA expressions in Th17 cells treated with or without recombinant mouse CD153 are shown. **(E)** A schematic illustration of the experimental protocol for retrovirus infection experiments. **(F)** Profiles of cells that performed TG2 knockdown using *Tgm2* shRNA (pLMP-sh*Tgm2*-GFP) and non-targeting control shRNA (pLMP-GFP) before and after sorting (left). mRNA expression of *Tgm2* was measured by qRT-PCR (right). **(G)** Profiles of cells that overexpressed TG2 using pMXs-*Tgm2*-hNGFR or introduced control pMXs-Mock- hNGFR (left). mRNA expression of *Tgm2* was measured by qRT-PCR (right). **(H)** mRNA expressions in CD30^hi^ Th2 cells, CD30^lo^ Th1 and Th17 cells, measured by qRT-PCR. Data are representative of at least two independent experiments.

Supplemental Figure 3 Characterization of CD4 T cells from *Tgm2*-deficient mice. **(A)** Representative CD4/CD8 profiles of thymocytes and splenocytes from *Tgm2*^+/+^ and *Tgm2*^−/−^ mice are shown. **(B)** Surface expressions of the indicated cell-surface marker molecules on splenic CD4 T cells from *Tgm2*^+/+^ (blue) and *Tgm2*^−/−^ (red) mice. **(C)** The percentages of cells in each cell division based on CFSE dilution are shown. **(D)** The profiles and the bar graph of IFNγ/IL-4 and IFNγ/IL-17A production in *in vitro* differentiated OTII Tg *Tgm2*^+/+^ and *Tgm2*^−/−^ Th1 or Th17 cells. The percentages of cells in each quadrant are indicated. **(E,F)** A schematic illustration of the experimental protocol for memory Th cell generation. **(G)** The absolute cell numbers of CD4^+^ Thy1.2^+^ OTII Tg Th2 cells in the indicated tissues one month after cell transfer **(E)**. Data are representative of at least two independent experiments. The mean values (4 mice per group) are shown. **(H)** A schematic illustration of the experimental protocol for *in vivo* memory CD4 T cell generation model for NP-specific antibody formation.

Supplemental Figure 4 Phenotypic characterization of *Tgm2*^fl/fl^ *Cd4Cre* Tg mice. **(A)** A schematic illustration of the experimental protocol for the memory Th17-dependent allergic airway inflammation model. **(B)** Representative CD4/CD8 profiles of thymocytes and splenocytes from *Tgm2*^fl/fl^ and *Tgm2*^fl/fl^ *Cd4Cre* mice are shown (left). Surface expressions of the indicated cell-surface marker molecules on splenic CD4 T cells from *Tgm2*^fl/fl^ WT (blue line) and *Tgm2*^fl/fl^*Cd4Cre* (red line) mice (right). **(C)** A schematic illustration of the experimental protocol for *in vivo* generated memory Th cells. **(D)** Representative profiles of CD154 (CD40L) expression and IFNγ production on CD4^+^CD44^hi^ splenocytes after stimulation with whole OVA.
