# Supplementary figures and images for: Essential Role for CD30-Transglutaminase 2 Axis in Memory Th1 and Th17 Cell Generation
Source: Front Immunol. 2020 Jul 21;11:1536. doi: 10.3389/fimmu.2020.01536 (PMC7385138; doi:10.3389/fimmu.2020.01536)

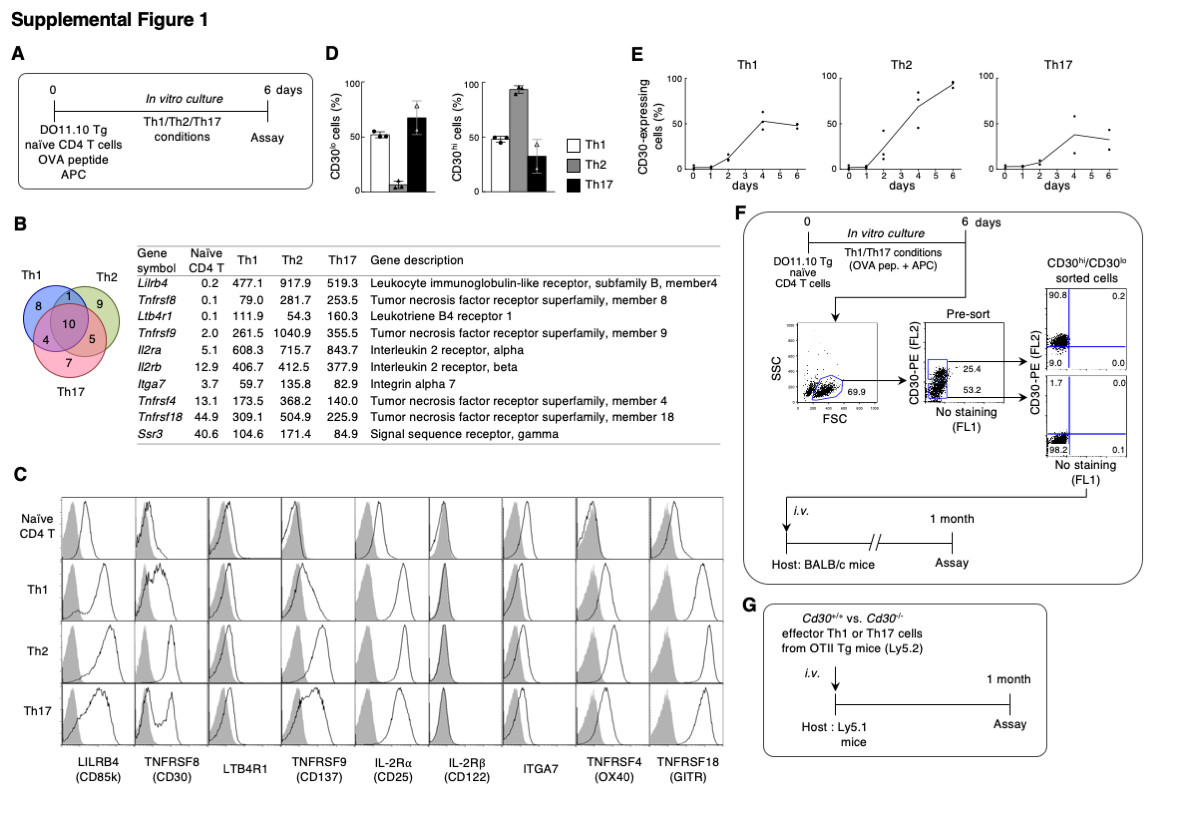

Supplement: Supplemental Figure 1 — CD30 expression in naïve CD4T, effector Th1, Th2 and Th17 cells. (A) A schematic illustration of the experimental protocol for in vitro-differentiated effector Th1, Th2, and Th17 cells. (B) Venn diagrams showing the overlap of “receptor” genes differentially expressed in Th1, Th2, and Th17 cells vs. naïve CD4 T cells. Gene expression in each Th cell population was measured by RNA-seq as FPKM (fragments per kilobase of exon per million mapped reads). Up-regulated genes were selected using the following criteria: (1) absolute FPKM > 50 in effector Th1, Th2, or Th17 cells, and (2) 2-fold change in the expression. “Receptor” (SP_PIR_Keyword) genes were selected using the DAVID analysis tool (http://david.abcc.ncifcrf.gov/home.jsp). The shared genes among Th1, Th2, and Th17 cells are listed (table). (C,D) Expression pattern of the indicated cell-surface marker molecules on naïve CD4 T cells, effector Th1, Th2, and Th17 cells 6 days after stimulation (C). The frequency of CD30hi and CD30lo Th1, Th2, and Th17 cells were shown (D). (E) The percentages of CD30-expressing cells during effector Th cell differentiation. DO11.10 Tg naïve CD4 T cells were stimulated as shown in (A), and the percentages of CD30-expressing cells were measured at days 0, 1, 2, 4, and 6. (F) A schematic illustration of the experimental protocol for memory Th cell generation by adoptive transfer of DO11.10 Tg effector Th1 or Th17 cells, including the gating and sorting strategy of CD30hi and CD30lo Th cells. (G) A schematic illustration of the experimental protocol for memory Th cell generation using OTII Tg Cd30+/+ or Cd30−/− naïve CD4 T cells (Ly5.2) Data are representative of at least two independent experiments (B–D). [file Image_1.tiff]

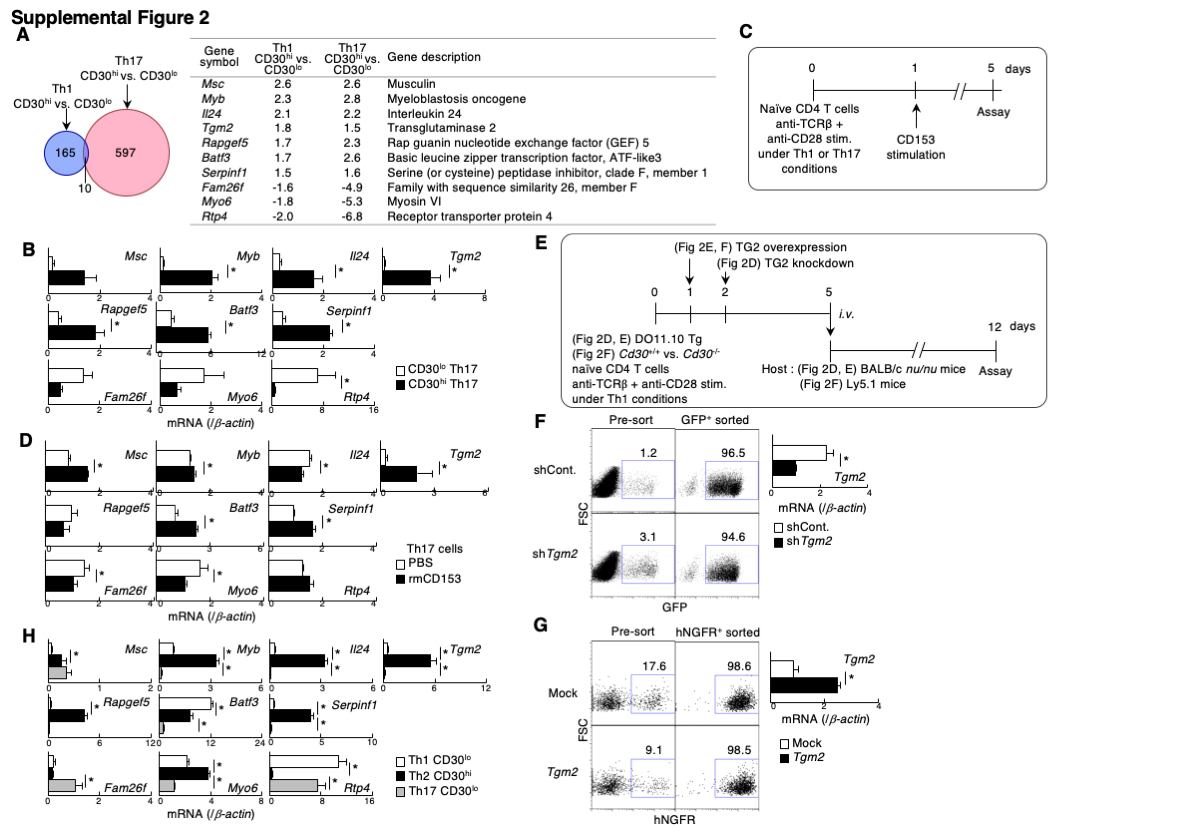

Supplement: Supplemental Figure 2 — Identification of the candidate genes that regulate memory Th1 and Th17 cell generation through CD30 signaling. (A) Venn diagrams of genes with changed expressions of a log2 value >1.5 between the CD30hi and CD30lo populations, measured by cDNA microarray analysis. Ten genes commonly changed expressions in Th1 and Th17 cells are listed (table). (B) mRNA expressions in CD30hi and CD30lo Th17 cells measured by qRT-PCR. (C) A schematic illustration of the experimental protocol for in vitro CD30 stimulation using recombinant mouse CD153 (CD30 ligand). (D) mRNA expressions in Th17 cells treated with or without recombinant mouse CD153 are shown. (E) A schematic illustration of the experimental protocol for retrovirus infection experiments. (F) Profiles of cells that performed TG2 knockdown using Tgm2 shRNA (pLMP-shTgm2-GFP) and non-targeting control shRNA (pLMP-GFP) before and after sorting (left). mRNA expression of Tgm2 was measured by qRT-PCR (right). (G) Profiles of cells that overexpressed TG2 using pMXs-Tgm2-hNGFR or introduced control pMXs-Mock- hNGFR (left). mRNA expression of Tgm2 was measured by qRT-PCR (right). (H) mRNA expressions in CD30hi Th2 cells, CD30lo Th1 and Th17 cells, measured by qRT-PCR. Data are representative of at least two independent experiments. [file Image_2.tiff]

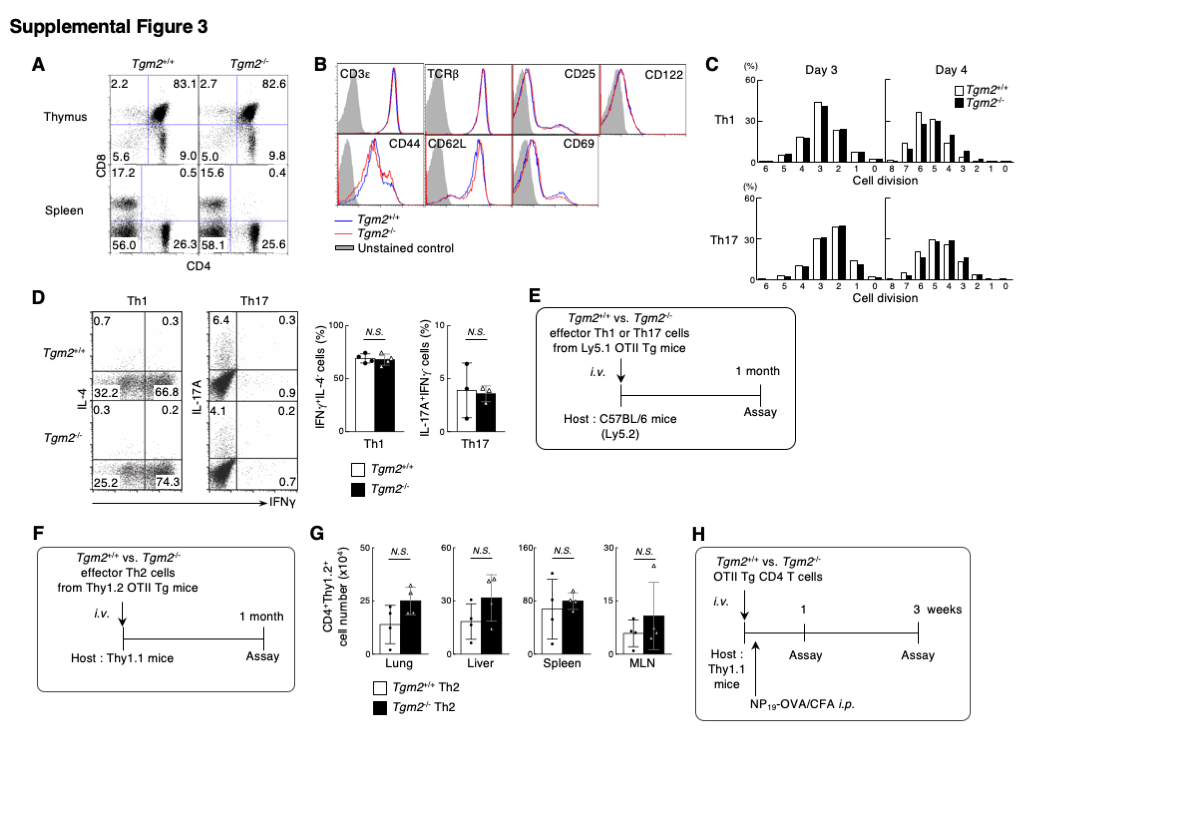

Supplement: Supplemental Figure 3 — Characterization of CD4 T cells from Tgm2-deficient mice. (A) Representative CD4/CD8 profiles of thymocytes and splenocytes from Tgm2+/+ and Tgm2−/− mice are shown. (B) Surface expressions of the indicated cell-surface marker molecules on splenic CD4 T cells from Tgm2+/+ (blue) and Tgm2−/− (red) mice. (C) The percentages of cells in each cell division based on CFSE dilution are shown. (D) The profiles and the bar graph of IFNγ/IL-4 and IFNγ/IL-17A production in in vitro differentiated OTII Tg Tgm2+/+ and Tgm2−/− Th1 or Th17 cells. The percentages of cells in each quadrant are indicated. (E,F) A schematic illustration of the experimental protocol for memory Th cell generation. (G) The absolute cell numbers of CD4+ Thy1.2+ OTII Tg Th2 cells in the indicated tissues one month after cell transfer (E). Data are representative of at least two independent experiments. The mean values (4 mice per group) are shown. (H) A schematic illustration of the experimental protocol for in vivo memory CD4 T cell generation model for NP-specific antibody formation. [file Image_3.tiff]

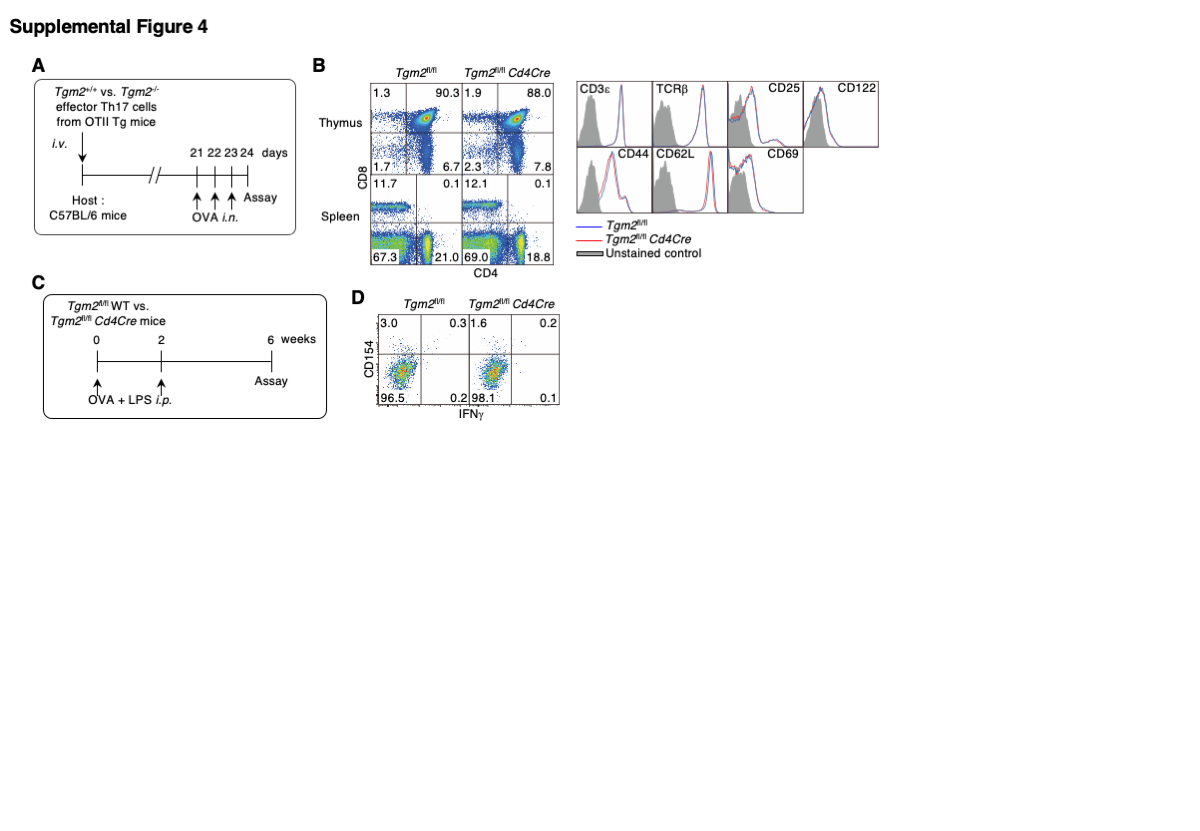

Supplement: Supplemental Figure 4 — Phenotypic characterization of Tgm2fl/fl Cd4Cre Tg mice. (A) A schematic illustration of the experimental protocol for the memory Th17-dependent allergic airway inflammation model. (B) Representative CD4/CD8 profiles of thymocytes and splenocytes from Tgm2fl/fl and Tgm2fl/fl Cd4Cre mice are shown (left). Surface expressions of the indicated cell-surface marker molecules on splenic CD4 T cells from Tgm2fl/fl WT (blue line) and Tgm2fl/flCd4Cre (red line) mice (right). (C) A schematic illustration of the experimental protocol for in vivo generated memory Th cells. (D) Representative profiles of CD154 (CD40L) expression and IFNγ production on CD4+CD44hi splenocytes after stimulation with whole OVA. [file Image_4.tiff]
