# Supplementary material for: Essential Role for CD30-Transglutaminase 2 Axis in Memory Th1 and Th17 Cell Generation
Source: Front Immunol. 2020 Jul 21;11:1536. doi: 10.3389/fimmu.2020.01536 (PMC7385138; doi:10.3389/fimmu.2020.01536)
Supplement: Supplementary file 5 [file Table_1.pdf]

Supplemental Table 1

Antibody	Clone
Anti-mouse CD3e	145-2C11
Anti-mouse CD4	RM4-5
Anti-mouse CD8a	53-6.7
Anti-mouse CD25	3C7
Anti-mouse CD30	mCD30.1
Anti-mouse CD44	IM7
Anti-mouse CD45.1 (Ly5.1)	A20
Anti-mouse CD45.2 (Ly5.2)	104
Anti-mouse CD62L	MEL-14
Anti-mouse CD69	H1.2F3
Anti-mouse CD85k	ZM4.1
Anti-mouse CD90.1 (Thy1.1)	OX-7
Anti-mouse CD90.2 (Thy1.2)	30-H12
Anti-mouse CD122	TM-b1
Anti-mouse CD137	1AH2
Anti-mouse CD153	RM153
Anti-mouse CD154	MR1
Anti-mouse CD357 (GITR)	DTA-1
Anti-mouse Integrin a7 (ITGA7)	FAB3518
Anti-mouse TCR DO11.10	KJ1.26
Anti-mouse Leukotriene B4 receptor 1 (LTB4R1)	Polycolonal
Anti-mouse CD134 (OX40)	OX-86
Anti-mouse TCRβ	H57-597
Anti-human CD271 (NGFR)	ME20.4
Anti-mouse IL-4	11B11
Anti-mouse IL-17A	TC11-18H10.1
Anti-mouse IFN-γ	XMG1.2
