# Supplementary material for: Essential Role for CD30-Transglutaminase 2 Axis in Memory Th1 and Th17 Cell Generation
Source: Front Immunol. 2020 Jul 21;11:1536. doi: 10.3389/fimmu.2020.01536 (PMC7385138; doi:10.3389/fimmu.2020.01536)
Supplement: Supplementary file 6 [file Table_2.pdf]

Supplemental Table 2

Primer	Sequence	Probe
β-Actin-L	ctaaggccaaccgtgaaaag	
β-Actin-R	accagaggcatacagggaca	#64
Batf3-L	agaaggctgacaagctccac	
Batf3-R	ccttcagcttcgaaatctcc	#1
Fam26f-L	agcctggtgaccctactgac	
Fam26f-R	actggaacaccactgaggaga	#7
Clca1 (Gob5)-L	aggaaaaccccaagcagtg	
Clca1 (Gob5)-R	gcaccgacgaacttgatttt	#46
Il24-L	agaaccagccaccttcacac	
Il24-R	gtgttgaagaaagggccagt	#75
Rapgef5-L	ctgcaggactaaccagtttgg	
Rapgef5-R	tggttgacataggacttcagctc	#68
Rtp4-L	gcaccagcagacagtgctt	
Rtp4-R	cctgagcagaggtccaactt	#47
Muc5ac-L	acttcaacggcagtccaaaa	
Muc5ac-R	ctcaaggggtgtcagcctaa	#55
Muc5b-L	accgtcacttgcctatcaaag	
Muc5b-R	gtagtgtgagtgcccccatc	#92
Msc-L	agctttccaaactggacacg	
Msc-R	gtccagagaccacgaatgg	#11
Myb-L	tgtcaacagagaacgagctga	
Myb-R	gctgcaagtgtggttctgtg	#40
Myo6-L	gatgctctaatggccaaaattaag	
Myo6-R	ttaacaagtgcatcatactctttctgt	#32
Serpinf1-L	ggactctgatctcaactgcaag	
Serpinf1-R	aagttctgggtcacggtcag	#4
Tgm2-L	ctcacgttcggtgctgtg	
Tgm2-R	tccctcctccacattgtca	#34
